# Comprehensive improvement in cardiac function following myosin inhibition after the ineffectiveness of myectomy and alcohol septal ablation: a case report

**DOI:** 10.1093/ehjcr/ytaf365

**Published:** 2025-08-14

**Authors:** Aleksandar Aleksandrov, Wilhelm Haverkamp, Adrian Constantin Borges, Thomas Zöller, Fabian Knebel

**Affiliations:** Department of Cardiology, Sana Klinikum Berlin Lichtenberg, Fanninger Strasse 32, Berlin 10365, Germany; Department of Cardiology, Sana Klinikum Berlin Lichtenberg, Fanninger Strasse 32, Berlin 10365, Germany; Department of Cardiology, Sana Klinikum Berlin Lichtenberg, Fanninger Strasse 32, Berlin 10365, Germany; Kardios Praxis Wittenbergplatz, Ansbacher Str. 17-19, Berlin 10787, Germany; Department of Cardiology, Sana Klinikum Berlin Lichtenberg, Fanninger Strasse 32, Berlin 10365, Germany

**Keywords:** Hypertrophic obstructive cardiomyopathy, Myosin inhibition, Case report, Echocardiography

## Abstract

**Background:**

Hypertrophic cardiomyopathy affects approximately 1 in 500 adults and is often associated with left ventricular outflow tract obstruction. Despite guideline-directed medical therapy and septal reduction procedures, a significant number of patients remain symptomatic. Recently, cardiac myosin inhibitors such as mavacamten have emerged as promising therapeutic options. By directly targeting the underlying hypercontractility, mavacamten can reduce left ventricular outflow tract gradients and improve functional capacity.

**Case summary:**

This report highlights the effects of myosin inhibition in a 72-year-old male patient with symptomatic hypertrophic obstructive cardiomyopathy. The patient was refractory to standard medical as well as interventional therapies (myectomy and septal ablation). Initiation of mavacamten therapy led to marked clinical improvement, including increased exercise capacity and weight loss due to improved physical activity. Echocardiography revealed regression of septal wall thickness, reduction of the left ventricular outflow tract gradient, and improvement of diastolic function. Furthermore, electrocardiogram changes consistent with hypertrophyincluding voltage criteria and T wave inversions—resolved completely. The cardiac biomarkers have normalized.

**Discussion:**

Myosin inhibition has the potency to reverse multiple pathophysiological mechanisms in hypertrophic obstructive cardiomyopathy—even in patients with advanced age and with long-standing, resistant, and symptomatic disease. The treatment effects could be observed rapidly.

Learning points

- Myosin inhibition is a promising therapeutic intervention for hypertrophic obstructive cardiomyopathy that appears to have a broad and significant impact on the clinical situation.
- Myosin inhibition reduces wall thickness (as measured by echocardiography and the Sokolow index in electrocardiograms), outflow gradients, improved diastolic left ventricular function, and cardiac biomarkers.

## Background

Hypertrophic cardiomyopathy (HCM) is a common inherited cardiomyopathy with global distribution.^1^ There are two types of HCM: a more common, which presents with dynamic left ventricular outflow tract (LVOT) obstruction, hypertrophic obstructive cardiomyopathy (HOCM), and a less common, without LVOT obstruction, hypertrophic non-obstructive cardiomyopathy. The HOCM is associated with symptoms such as dyspnoea, chest pain, and syncope, and if undiagnosed or insufficiently treated, it can lead to progressive heart failure or sudden cardiac death (SCD). Standard therapy includes beta-blockers, verapamil, or disopyramide; in refractory cases, septal reduction via myectomy or alcohol septal ablation may be indicated. However, not all patients respond adequately to these interventions. Mavacamten, a first-in-class cardiac-specific myosin inhibitor, addresses the underlying pathophysiology of HOCM and has shown promising results in reducing LVOT gradients and improving symptoms.^2^ We present a case of a patient with symptomatic HOCM refractory to both medical and interventional therapy who showed marked improvement with mavacamten.

## Summary figure

**Figure:**
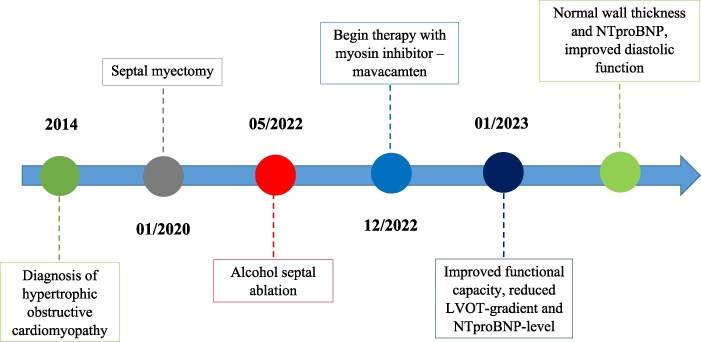


## Case summary

A 72-year-old Caucasian male with HOCM was referred to our cardiology outpatient department. The patient suffered from a significant shortness of breath [New York Heart Association (NYHA) Class III] and angina symptoms which were refractory to standard medical therapy including calcium channel blockers (verapamil was discontinued because of worsening the shortness of breath), beta-blockers, an angiotensin receptor blocker, a mineralocorticoid receptor antagonists, and diuretics.

Co-morbidities included arterial hypertension, diabetes mellitus type 2 (under dietary control), obesity, dyslipidaemia, and obstructive sleep apnoea syndrome with mask ventilation. He is a never-smoker. There was no family history of cardiomyopathy or SCD.

The patient was initially diagnosed with HOCM at the age of 64 in 2014 following a transthoracic echocardiography with a maximal LVOT pressure gradient of 60 mmHg at rest (normal resting LVOT gradient is <30 mmHg). A coronary angiography, performed in 2011, excluded a coronary artery disease. Cardiac magnetic resonance imaging (CMR) confirmed the typical features of LVOT obstruction. A stress CMR (2015) revealed flow acceleration in the LVOT, asymmetric severe left ventricular hypertrophy (basal inferoseptal wall 19 mm, basal inferolateral wall 11 mm), and intramural fibrosis (inferior and inferoseptal).

In 2020, worsening of shortness of breath and chest pain upon exertion and a peak LVOT gradient of 94 mmHg triggered a surgical *trans*-aortic septal myectomy (Morrow procedure). Typically, this procedure results in a >90% reduction in gradient and marked symptom relief. However, the symptoms persisted, and the post-operative Valsalva gradient remained elevated (47 mmHg).

Due to worsening of the symptoms and an increase in LVOT gradient to >70 mmHg at rest, alcohol septal ablation targeting the first septal perforator was performed in 2022. Although a transient CK elevation (11.2 µkat/L) indicated effective myocardial necrosis, there was no sustained reduction in the LVOT gradient or improvement in clinical status.

### Physical examination and diagnostic assessment

Physical examination revealed a split-second heart sound, S4 heart sound, a systolic 3/6 grade parasternal murmur in the third intercostal space, and laterally displaced apical precordial impulse. The patient had an elevated body mass index. A 12-lead electrocardiogram (ECG) showed a sinus rhythm with left axis deviation, anterolateral ST segment depression, T wave inversion, and voltage criteria for left ventricular hypertrophy, first degree AV block, and complete right bundle branch block. The patient had an unremarkable 24-h ECG monitor with no evidence of ventricular tachycardia. The echocardiography showed left ventricular (LV) hypertrophy of 23 mm (septal) with LVOT obstruction with a pressure gradient of 54 mmHg at rest and 95 mmHg during Valsalva manoeuvre. The baseline echocardiogram showed diastolic dysfunction (pseudonormal pattern) and no mitral regurgitation. The European Society of Cardiology 5-year SCD risk score was calculated at 2.03%. The primary preventive implantation of an implantable cardioverter-defibrillator was not indicated.

Given the failure of maximal medical therapy and two septal reduction interventions, the patient was deemed suitable for myosin inhibition with mavacamten. The indication was reviewed and approved under the compassionate use programme of the manufacturer (Bristol Myers Squibb).

### Intervention and follow-up

**Figure 1:**
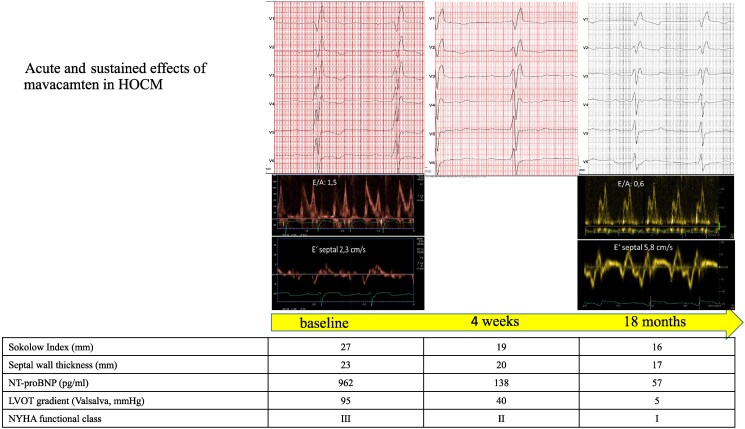
Acute and sustained effects of mavacamten in the hypertrophic obstructive cardiomyopathy patient. LVOT, left ventricular outflow tract; NYHA, New York Heart Association.

Oral therapy with the new myosin inhibitor mavacamten (5 mg OD) was started in December 2022. Four weeks later, the functional capacity started to improve. The LVOT gradient, wall thickness, N-terminal pro b-type natriuretic peptide (NT-proBNP), Sokolow index, and NYHA class are shown in *Figure 1*. However, the acoustic window was initially reduced because of obesity. Interestingly, the image quality improved significantly over time after the therapy with mavacamten and a relevant weight loss to increased physical activity. After 18 months of therapy with mavacamten, there was a normalization of NT-proBNP, a regression of the signs of LV hypertrophy in the ECG. In addition to a reduction of the Sokolow index, we observed a resolution of the T wave inversion of leads V3–V6. Moreover, the diastolic function has improved from pseudonormalization to impaired relaxation as shown in *Figure 1*. The patient has reported that he has lost 15 kg of body weight due to the markedly increased physical activity.

Transthoracic echocardiography and clinical evaluation have been performed 4, 8, and 12 weeks after the first dose of mavacamten and then every 12 weeks afterwards until a maintenance dose has been achieved. After that, follow-up including echocardiography assessment and clinical evaluation has been done every 6 months.

## Discussion

Our case illustrates the significant acute and long-term clinical and objective improvements in a HOCM patient treated with mavacamten. Mavacamten is a new treatment option for symptomatic patients with symptomatic HOCM. It is a first-in-class, small molecule, selective allosteric inhibitor of cardiac myosin ATPase specifically developed to target the underlying pathophysiology of HCM by reducing actin–myosin cross-bridge formation, thereby reducing contractility and improving myocardial energetics. In two Phase 3 double-blind, placebo-controlled, multicentre randomized clinical trials (EXPLORER-HCM and VALOR-HCM), the treatment with mavacamten successfully relieved LVOT gradients; improved symptoms, exercise performance, and health status; and significantly reduced the fraction of patients meeting guideline criteria for septal reduction therapy.^2,3^ The LV hypertrophy reduction was already demonstrated by CMR assessment in the EXPLORER-HCM study change in LV mass index: −17.4 g/m^2^ in the mavacamten group vs. −1.6 g/m^2^ in the placebo group (*P* < 0.0001)^2^ and in another smaller study.^4^ The long-term safety and efficacy of mavacamten were demonstrated in the MAVA-LTE study.^5^

Unlike septal reduction therapies, mavacamten does not only address the regional problem of LVOT obstruction. Myosin inhibition in principle leads to a global regression of LV hypertrophy. This is reflected by a decrease of wall thickness in echo, regression of LV hypertrophy in 12-lead ECG, and normalization of the cardiac biomarker NT-proBNP. In summary, our case underlines the global effects of myosin inhibition in HOCM not only on the outflow tract obstruction as seen in other studies.^2,3^

A recent case study has examined the beneficial effects of mavacamten on the electromechanical dispersion after 3 years of treatment. Mechanical dispersion of the QTc interval is seen as a marker of arrhythmogenic risk in HOCM patients.^6^

The holistic approach of myosin inhibition in HOCM with mavacamten seems to give rise to many relevant scientific questions: Should mavacamten be taken temporarily until the regression of LV hypertrophy or for unlimited time? What about dose adjustments? Can it be used in symptomatic HCM with mid-ventricular obstruction or without any inducible pressure gradient in the LVOT? Will it reduce mortality in the long-term follow-up? Further studies are necessary to answers these questions.

In summary, myosin inhibition with mavacamten represents a promising therapeutic option for patients with symptomatic HOCM refractory to standard medical and invasive treatments. In this case, the therapy with mavacamten led to a marked improvement in functional status, normalization of cardiac biomarkers (NT-proBNP), regression of septal hypertrophy and the LVOT gradients, and resolution of ECG signs of LV hypertrophy. These findings underscore the broad, multimodal benefits of myosin inhibition in advanced obstructive HCM.

## Data Availability

Data will be available on request.

## Lead author biography

Internist and cardiologist, Fellow of the European Society of Cardiology (FESC), and Head of the Department of Internal Medicine/Cardiology at the Sana Klinikum Berlin Lichtenberg, Germany, with an affiliation to the Deutsches Herzzentrum der Charité (DHZC), Berlin, Germany.

**Consent:** The patient has given his consent to this case report in written and is compliant with the COPE guidelines.

**Funding:** None declared.
